# Supplementary material for: A sex difference in the response of the rodent postsynaptic density to synGAP haploinsufficiency
Source: eLife. 2020 Jan 15;9:e52656. doi: 10.7554/eLife.52656 (PMC6994236; doi:10.7554/eLife.52656)
Supplement: Supplementary file 1. — The Table shows the steps to calculate a synGAP/PSD-95 ratio and a TARPs/PSD-95 ratio from digital data recorded from one pair of gels containing samples from animals 33 and 34 (see Figure 2—figure supplement 1). Data were collected from at least three technical replicates of synGAP gel lanes for each animal and usually six technical replicates of target gel lanes. The resulting ratios were averaged. [file elife-52656-supp1.pdf]

Supplementary File 1. Example calculations of synGAP/PSD-95 ratio and TARPs/PSD-95 ratio for animals 33 and 34.

The Table shows the steps to calculate a synGAP/PSD-95 ratio and a TARPs/PSD-95 ratio from digital data recorded from one pair of gels containing samples from animals 33 and 34 (see Figure 1-figure supplement 1). Data were collected from at least 3 technical replicates of synGAP gel lanes for each animal and usually 6 technical replicates of target gel lanes. The resulting ratios were averaged.

Animal ID	Signal	Intensity	Signal Intensity - Bkg.	synGAP/PSD-95 Ratio
33	PSD95	6620000	2540000	0.489
33	PSD95_Background	4080000		
33	SynGAP	1720000	1242000	
33	SynGAP_Background	478000		
34	PSD95	5800000	1970000	0.686
34	PSD95_Background	3830000		
34	SynGAP	1800000	1351000	
34	SynGAP_Background	449000		
**Animal ID**	**Signal**	**Intensity**	**Signal Intensity - Bkg.**	**TARPs/PSD-95 Ratio**
33	PSD95	5460000	2560000	0.104
33	PSD95_Background	2900000		
33	TARPs	1170000	265000	
33	TARPs_Background	905000		
34	PSD95	3880000	1760000	0.092
34	PSD95_Background	2120000		
34	TARPs	853000	162000	
34	TARPs_Background	691000		
